# Cellular senescence in brain aging and cognitive decline

**DOI:** 10.3389/fnagi.2023.1281581

**Published:** 2023-11-23

**Authors:** Areez Shafqat, Saifullah Khan, Mohamed H. Omer, Mahnoor Niaz, Ibrahem Albalkhi, Khaled AlKattan, Ahmed Yaqinuddin, Tamara Tchkonia, James L. Kirkland, Shahrukh K. Hashmi

**Affiliations:** ^1^College of Medicine, Alfaisal University, Riyadh, Saudi Arabia; ^2^Medical College, Aga Khan University, Karachi, Pakistan; ^3^School of Medicine, Cardiff University, Cardiff, United Kingdom; ^4^Robert and Arlene Kogod Center on Aging, Mayo Clinic, Rochester, MN, United States; ^5^Department of Internal Medicine, Mayo Clinic, Rochester, MN, United States; ^6^Clinical Affairs, Khalifa University, Abu Dhabi, United Arab Emirates; ^7^Department of Medicine, SSMC, Abu Dhabi, United Arab Emirates

**Keywords:** cellular senescence, aging, cognitive decline, therapy-induced senescence (TIS), obesity, traumatic brain injury, microglia senescence, astrocyte senescence

## Abstract

Cellular senescence is a biological aging hallmark that plays a key role in the development of neurodegenerative diseases. Clinical trials are currently underway to evaluate the effectiveness of senotherapies for these diseases. However, the impact of senescence on brain aging and cognitive decline in the absence of neurodegeneration remains uncertain. Moreover, patient populations like cancer survivors, traumatic brain injury survivors, obese individuals, obstructive sleep apnea patients, and chronic kidney disease patients can suffer age-related brain changes like cognitive decline prematurely, suggesting that they may suffer accelerated senescence in the brain. Understanding the role of senescence in neurocognitive deficits linked to these conditions is crucial, especially considering the rapidly evolving field of senotherapeutics. Such treatments could help alleviate early brain aging in these patients, significantly reducing patient morbidity and healthcare costs. This review provides a translational perspective on how cellular senescence plays a role in brain aging and age-related cognitive decline. We also discuss important caveats surrounding mainstream senotherapies like senolytics and senomorphics, and present emerging evidence of hyperbaric oxygen therapy and immune-directed therapies as viable modalities for reducing senescent cell burden.

## Introduction

Increasing life expectancy and a declining birth rate, especially in the West, have led to an aging population at higher risk of age-related chronic diseases that incur significant morbidity, mortality, and healthcare expenditures (Cutler et al., 1990). Consequently, medical research on promoting healthy aging has become an essential area of investigation carrying significant public health and economic implications (Ferrucci et al., 2020).

The mechanisms underlying brain aging have garnered significant attention due to the significant number of patients suffering from dementia and Alzheimer’s disease (AD). The cost of managing these patients exceeds that of cancer and cardiovascular disease patients combined (Kukull et al., 2002; Hebert et al., 2013). Importantly, however, cognitive decline is observable in individuals without AD or overt neurodegenerative changes (Gonzales et al., 2022).

Age-related mild cognitive impairment (MCI) and late-onset AD can be mechanistically explained by processes governing biological aging. Currently, 12 biological aging hallmarks have been identified: genomic instability, telomere attrition, epigenetic alterations, loss of proteostasis, altered nutrient sensing, mitochondrial dysfunction, stem cell exhaustion, altered intracellular communication, cellular senescence, disabled macroautophagy, chronic inflammation (i.e., inflammaging), and gut microbiome dysbiosis (López-Otín et al., 2013, 2023). The geroscience hypothesis posits that age-related diseases arise from the cumulative effects of these biological aging hallmarks and that targeting them constitutes an avenue to ameliorate age-related diseases (Kennedy et al., 2014).

Cellular senescence describes a state of cell cycle arrest accompanied by characteristic morphological, cellular, and molecular changes (Zhang L. et al., 2022). Studies using pharmacological targeting of senescent cells (SCs), transplanting SCs, and transgenic mouse models have demonstrated a causal relationship between SC accumulation and age-related tissue dysfunction, with addition of SCs being shown to accelerate aging phenotypes on the one hand and clearance being shown to alleviate them on the other (Xu et al., 2015; Tchkonia and Kirkland, 2018; Xu et al., 2018; Kirkland and Tchkonia, 2020; Wang et al., 2020a; Xu et al., 2021; Chaib et al., 2022; Sun et al., 2022; Zhang et al., 2023). In the brain, SCs become more abundant with aging in mice, which is associated with cognitive decline, and their depletion mitigates neuroinflammation and delays cognitive decline (Ogrodnik et al., 2021; Zhang X. et al., 2022).

This review explores the association between cellular senescence and age-related cognitive decline. We also discuss how cellular senescence may underlie cognitive decline in different patient populations that exhibit a premature brain aging phenotype. These patients include cancer survivors, traumatic brain injury (TBI) patients, obese individuals, obstructive sleep apnea (OSA) patients, and chronic kidney disease (CKD) patients. Understanding the role of senescence in cognitive decline is essential, especially considering the rapidly evolving field of senotherapeutics. Targeting SCs could mitigate early brain aging and reduce a significant burden on patients, healthcare systems, and society.

## Cellular senescence and senotherapies

**Figure 1:**
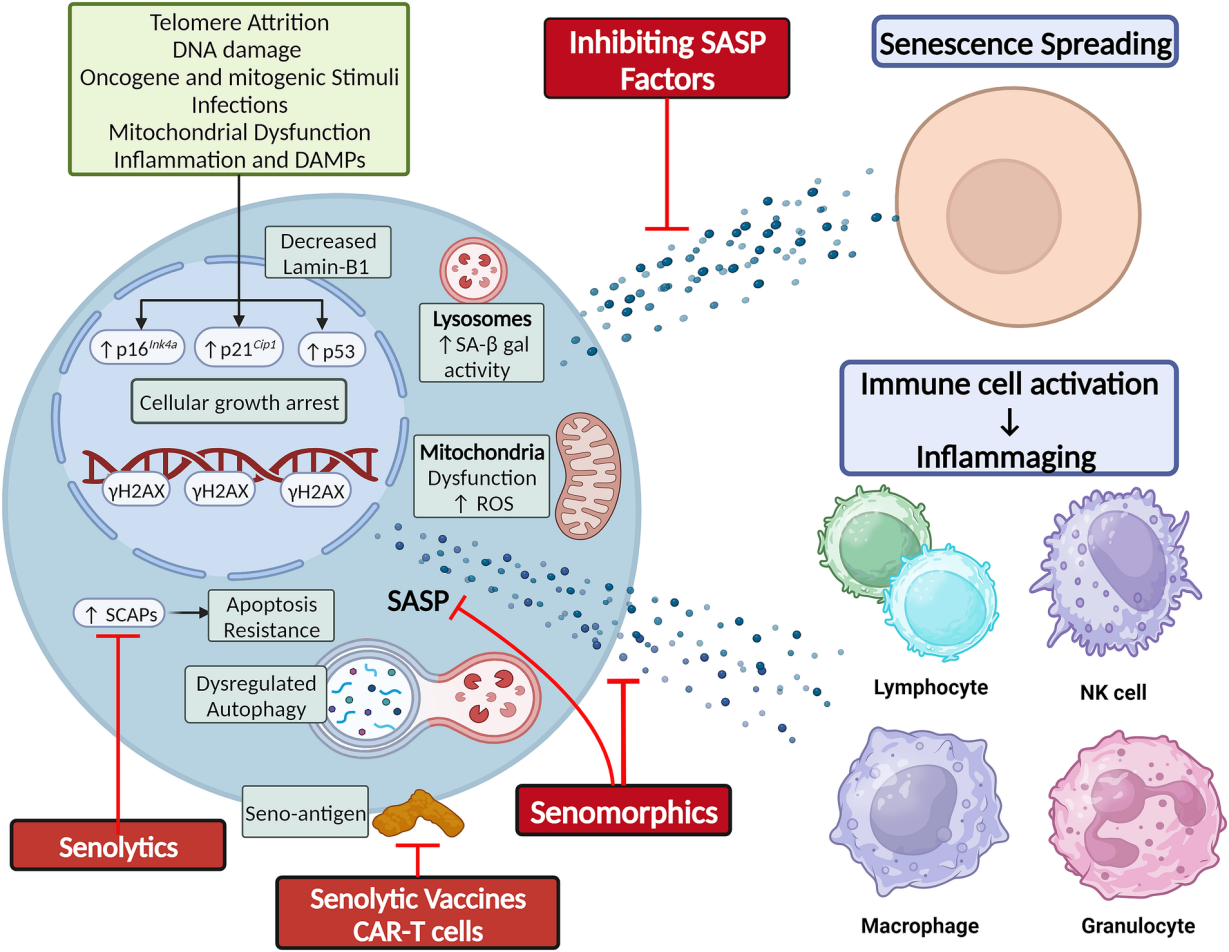
Cellular senescence is classically secondary to telomere shortening and DNA damage, but can also result from other cellular stressors. Hallmarks of senescence include elevated SA-β-gal activity, reduced lamin-B1 expression, mitochondrial dysfunction and elevated ROS production, apoptosis resistance by upregulation of SCAPs, and elaboration of a SASP composed of cytokines, chemokines, and growth factors. The SASP mediates the non-cell autonomous effects of senescence, including senescence-spreading and activating innate and adaptive immune cells to foster chronic low-grade inflammation. Figure was created using BioRender.com.

Cellular senescence is a state of cell cycle arrest originally described in 1961 by Hayflick and Moorehead when they observed that cultured human fibroblasts stopped dividing after 40–60 serial cell divisions (Hayflick and Moorhead, 1961). This phenomenon was due to telomere shortening, which triggered a DNA damage response (DDR) and induced replicative senescence (Olovnikov, 1973; Olovnikov, 1996). Senescence induction has now been linked to various other stimuli, including epigenetic alterations, oxidative stress, mitochondrial dysfunction, inactivation of tumor suppressor genes, mechanical or shear stress, pathogens, and activation of oncogenes (Figure 1; Tchkonia et al., 2013; Gorgoulis et al., 2019; Tripathi et al., 2021a).

Senescence induction in response to these various stimuli is orchestrated by the p53/p21*^Cip1/Waf1^* axis, p16*^Ink4a^*/Rb axis, and other mechanisms. The proteins involved in these pathways can serve as markers of senescence. Other features of SCs can be structural changes (flattened and enlarged cellular morphology), DNA and nuclear changes (DDR foci and decreased Lamin-B1 expression), elevated lysosomal enzyme senescence-associated β-galactosidase (SA β-gal) active at pH 6, mitochondrial changes [impaired membrane integrity, increased reactive oxygen species (ROS) production], upregulation of senescence-associated anti-apoptotic pathways (SCAPs), and elaboration of a senescence-associated secretory phenotype (SASP) that can comprise cytokines, chemokines, growth factors, proteases, bioactive small molecules, and nucleotides (e.g., microRNAs and mitochondrial DNA) (Figure 1; Gorgoulis et al., 2019; Iske et al., 2020; Tripathi et al., 2021b; Zhang et al., 2021; Nunes et al., 2022). While these changes can be used to identify SCs, no single specific marker of SCs is currently agreed upon, and more sensitive and specific markers of SC burden are needed. To this end, a gene set of 125 senescence-associated genes called SenMayo was recently used to identify SCs across multiple tissues in both bulk and single-cell RNA sequencing (scRNA-seq) data. The genes comprising SenMayo were shown to increase with aging and be responsive to SC clearance in transgenic mice (Saul et al., 2022).

### Senescence in aging

Cellular senescence is a double-edged sword. It induces growth arrest in potentially tumorigenic cells (Schosserer et al., 2017). It also plays essential roles in normal embryogenesis (Storer et al., 2013; Lorda-Diez et al., 2015) and wound healing (Demaria et al., 2014; Da Silva-Álvarez et al., 2020). However, the chronic accumulation of SCs in tissues leads to tissue dysfunction and chronic disease (Biran et al., 2017; Xu et al., 2017). To explain this, SCs utilize their SASP to exert cell-autonomous effects, reinforcing their own senescent phenotype through autocrine effects, and non-cell-autonomous effects, inducing senescence in neighboring as well as distant cells (Acosta et al., 2013; Xu et al., 2018). Additionally, the spillover of SASP factors into the circulation fosters a chronic low-grade inflammatory response that promotes age-related phenotypes (Figure 1; Acosta et al., 2013; Xu et al., 2018).

These pro-aging effects of SCs are made apparent when transplanting SCs into healthy mice, which accelerates the aging process and results in early death (Baker et al., 2011; Xu et al., 2018). SCs are also physically present at sites of chronic diseases, such as osteoarthritis, idiopathic pulmonary fibrosis (IPF), cataracts, age-related macular degeneration, atherosclerosis, sarcopenia, renal dysfunction, dementias, and organ transplant dysfunction (Chaib et al., 2022). Mouse models in which highly p16*^Ink4a^*-or p21*^Cip1/Waf1^*-expressing cells can be systemically depleted upon administration of agents that have little to no effects in wild-type mice have been developed (Wang et al., 2021; Chandra et al., 2022). These models have been utilized to systemically deplete SCs and have helped to establish a causal relationship between senescence and age-related chronic diseases (Baker et al., 2011; Demaria et al., 2014; Kirkland and Tchkonia, 2017).

### Senolytics

A pioneering study by Zhu et al. (2015) leveraged the observation that SCs upregulate SCAPs to suggest a senolytic approach. This involves using drugs that inhibit SCAPs to selectively induce apoptosis in SCs (i.e., senolysis). Dasatinib, a chemotherapeutic agent, and quercetin, a naturally occurring flavonoid, were the first discovered senolytics. These drugs selectively depleted SCs by targeting SCAPs and reduced senescence markers in the leg muscle and inguinal fat of irradiated mice, thereby restoring exercise capacity and endurance to levels comparable to control non-irradiated mice (Zhu et al., 2015). Fiscetin is also a naturally occurring flavonoid closely related to quercetin that has senolytic properties (Yousefzadeh et al., 2018). Navitoclax (ABT-263) is an inhibitor of BCL-2, conferring to it its senolytic effect. However, unlike dasatinib and quercetin, navitoclax targets a specific SCAP and not SCs specifically, leading to off-target side effects like thrombocytopenia that are dose-limiting (Chang et al., 2016; Zhu et al., 2016). These four drugs were the first-generation senolytics. Subsequent studies identified numerous senolytics through the original hypothesis-driven SCAP-targeting approach, serendipity, and conventional high-throughput library screens (Chang et al., 2016; Fuhrmann-Stroissnigg et al., 2017; Zhu et al., 2017; Fuhrmann-Stroissnigg et al., 2019; Xu et al., 2021; Samakkarnthai et al., 2023). UBX1235 like navitoclax is an anti-BCL senolytic, but is tailored to targeting senescence-related disorders in the eye and shown tolerability and encouraging efficacy in phase I and II clinical studies on age-related macular degeneration (Hassan and Bhatwadekar, 2022) (NCT05275205). A major advantage of senolytic drugs is their “hit-and-run” principle of administration, whereby intermittent administration is as effective as continuous administration, since senescence induction takes time and SCs are present only in small numbers in tissues (Kirkland et al., 2017). The timeline of senolytic drug discovery, i.e., their evolution from benchwork to clinical trials, has recently been reviewed (see Chaib et al., 2022).

A recent phase I, single-blinded, single-center, randomized, placebo-controlled study showed that the combination of dasatinib and quercetin was generally tolerable and safe in 12 idiopathic pulmonary fibrosis (IPF) patients (Nambiar et al., 2023). Mild side effects were higher in the treatment group and were generally those associated with the chemotherapeutic drug dasatinib, such as nausea, weakness, headache, feeling unwell and sleep disturbances (Nambiar et al., 2023).

There are several ongoing trials to investigate the role of senolytics in the prevention or progression modulation of neurodegenerative diseases. These trials include ALSENLITE (NCT04785300) and SToMP-AD (NCT04063124; NCT04685590). Preliminary reports from the Phase I SToMP-AD trial of five participants exploring the use of dasatinib + quercetin, as a potential treatment for AD were recently reported (Orr et al., 2023). Blood levels of the compounds increased in all participants with detectable levels in the cerebrospinal fluid (CSF), and no reported adverse events. While cognitive and neuroimaging measures did not significantly change post-treatment, CSF levels of certain SASP-typical cytokines/chemokines (IL-17E, IL-21, IL-23, IL-17A/F, IL-17D, IL-10, VEGF, IL-31, MCP-2, MIP-1β, and MIP-1α) were significantly decreased in treated patients (Gonzales et al., 2023; Orr et al., 2023).

### Senomorphics

Also known as senostatics, senomorphic drugs modulate senescence markers or attenuate SASP components to achieve effects similar to senolytics, but without causing apoptotic cell death (Lagoumtzi and Chondrogianni, 2021). Some of these agents act on transcriptional regulators of the SASP, such as the ATM, mTOR, JAK/STAT, and FOXO (Zhang et al., 2023). Among senomorphic drugs are rapamycin, metformin, and resveratrol (Zhang et al., 2023). Other compounds, such as procyanidin C1 or intravenous zoledronic acid, can exhibit both senomorphic and senolytic properties (Xu et al., 2021; Samakkarnthai et al., 2023). Preclinical research indicates that some senomorphic drugs can prolong healthspan of mice and decrease the incidence of various age-related pathologies, including cancers, cardiovascular diseases, metabolic disorders, cognitive decline, and neurodegenerative diseases (Zhang et al., 2023).

## Cellular senescence in the aging brain

**Figure 2:**
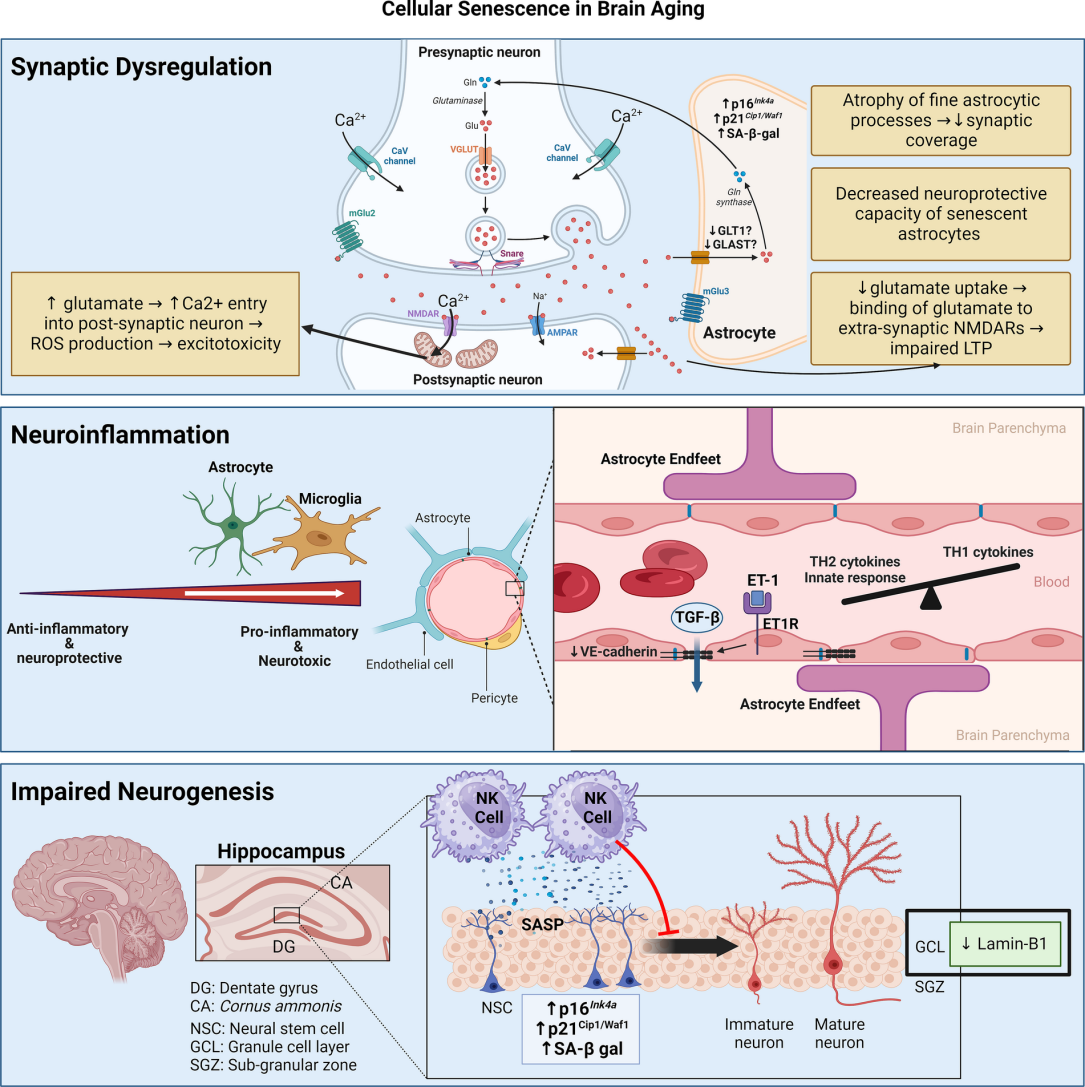
Senescence contributes to brain aging through three main mechanisms: synaptic dysfunction, neuroinflammation, and impaired neurogenesis. Synaptic dysfunction can be due to senescence of astrocytes, which decrease neurotransmitter uptake and display decreased neuroprotective capacity. Neuroinflammation stems from senescence of astrocytes and microglia, skewing them towards pro-inflammatory phenotypes. Senescence of cerebrovascular endothelial cells and pericytes may contribute to BBB disruption and consequent leakage of factors like TGF-β, which can induce astrocyte senescence. Senescence of neuroblasts/neuronal stem cells can activate neurotoxic NK cell functions which, in turn, eliminate neuroblasts, thereby impairing neurogenesis. Figure was created using BioRender.com.

Biologically, the accumulation of SCs in the brain has three main consequences: neuroinflammation, impaired neurogenesis, and synaptic dysfunction due to astrocyte senescence (Figure 2).

### Neuroinflammation

An important feature of SCs is their ability to secrete a composite of pro-inflammatory cytokines and chemokines. These mediators foster a chronic low-grade inflammatory response called inflammaging, which is causally linked to the aging process in the brain—termed neuro-inflammaging (Franceschi et al., 2000; Franceschi and Campisi, 2014). In general, neuro-inflammaging involves the skewing of the immune system towards activation of innate immunity and T-helper 2 (TH_2_) responses (Giunta et al., 2008; Hu et al., 2019). Diseases like multiple sclerosis and AD accentuate this trend toward non-TH_1_ cytokines (Hu et al., 2019). In the CSF, levels of several cytokines (e.g., IL-1β, IL-4, IL-6, IL-8, IFNγ, G-CSF, and GM-CSF) are negatively associated with AD-related progression of cognitive decline, suggesting a protective function of these factors (Taipa et al., 2019; Albrecht et al., 2021). Peripheral inflammaging features elevations in mediators like IL-1β, IL-6, TNFα, and CRP, have been linked to the progression of age-related cognitive decline and the progression of neurodegenerative disease-related dementia (Feng et al., 2023). A different set of circulating inflammatory proteins like IFNγ and IL-12, which are hallmarks of a TH_1_ response, are seemingly protective against cognitive decline (Yang et al., 2022).

The mediators of neuro-inflammaging are thought to be derived from glial cells such as astrocytes and microglia (Allen et al., 2023). This is coupled with aging-related blood–brain barrier (BBB) disruption from dysfunction of the cerebral microvasculature, which further propagates neuroinflammation by making the normally immune-privileged brain more susceptible to peripheral immune cell infiltration as well as the circulating inflammaging mediators (Watanabe et al., 2020). Studies have demonstrated that age-related senescence in astrocytes and microglia results in the elaboration of a pro-inflammatory SASP (Chinta et al., 2015; Sikora et al., 2021; Zhang X. et al., 2022), while endothelial cell and pericyte senescence of the cerebral microvasculature has been linked to BBB disruption (Yamazaki et al., 2016; Iwao et al., 2023). Cellular senescence thus emerges as a key hypothesis that underlies neuro-inflammaging, central to age-related cognitive deterioration with or without neurodegeneration (Scheiblich et al., 2020).

Regarding vascular senescence, in naturally aged 28-month-old C57BL/6 mice (roughly equivalent to a 75-year-old human), the proportion of senescent brain microvascular endothelial cells is 10% higher compared to 3-month-old mice (Kiss et al., 2020). In genetically modified BubR1-hypomorphic (BubR1^H/H^) mice, which are progeroid models displaying accelerated aging phenotypes, endothelial cells and pericytes of the cerebral vasculature display increased SA β-gal and p16*^Ink4a^* activity, which is associated with reduced gap junction coverage in cerebral micro-vessels and increased BBB permeability (Yamazaki et al., 2016). Endothelin-1 (ET1)—a potent vasoconstrictor that is upregulated with aging (Donato et al., 2009)—binds the ET-A receptor on brain microvascular endothelial cells, leading to an upregulation of senescence markers and a downregulation of the adherens junction protein VE-cadherin (Abdul et al., 2022). Senescence induction in serially passaged pericytes or those isolated from aged rat brains increase permeability of *in vitro* BBB models composed of intact BMECs (Iwao et al., 2023). However, causal links between vascular senescence and age-related cognitive decline require assessing whether genetic or pharmacologic modulation of senescence in endothelial cells or pericytes mitigates this phenotype.

Regarding astrocytes, Clarke et al. (2018) carried out transcriptional profiling of astrocytes through the mouse lifespan and observed an age-dependent skewing of astrocyte transcriptome towards a pro-inflammatory state, likely under the influence of microglia-derived pro-inflammatory signals like IL-1, TNF-α, and C1q. Senescence markers like SA β-gal-positivity, lamin-B1 downregulation, mitochondrial dysfunction, and increased ROS production are upregulated in aged astrocytes, associated with the elaboration of a pro-inflammatory SASP reminiscent of neurotoxic/proinflammatory (formerly called A1) astrocytes (Liddelow et al., 2017; Matias et al., 2022). Human astrocytes made senescent by serial replication elaborate a SASP composed of IL-8, GM-CSF, angiogenin, MMP-3, MMP-10 and TIMP-2, while the production of anti-inflammatory cytokines like IL-10 is reduced (Lye et al., 2019). On a molecular level, senescent astrocytes display a significant dysregulation in their expression of proteins involved in splicing of mRNA transcripts, which may allow them to alter their proteome with respect to the SASP (Lye et al., 2019). Importantly, mRNA transcripts (e.g., p14^ARF^, GFAP-α, and TAU3) upregulated by senescent astrocytes have been associated with cognitive decline in an aging population (Lye et al., 2019). What triggers astrocyte senescence in the aging brain was recently investigated. Serum albumin leaks into the brain parenchyma due to age-related BBB dysfunction, activates the TGF-β receptor-II on astrocytes, and induces senescence both *in vitro* and *in vivo* (Preininger et al., 2023). TGF-β-induced senescent hippocampal astrocytes exhibit increased mRNA levels of SASP components such as TGF-β1, IL-1β, IL-6, CXCL10, MCP-1, and CCL5 (RANTES), which is preventable by genetic knockdown or pharmacologic blockade of the astrocyte TGF-β receptor (Preininger et al., 2023). Whether modulation of TGF-β-related astrocyte senescence can influence cognitive health remains investigational.

Regarding microglia, there is no age-associated brain-wide shift in their phenotype towards inflammation, but subsets of inflammatory microglia do appear in the aged mouse brain, likely driven by local cues like BBB disruption or microinfarcts (Hammond et al., 2019a). The hippocampi of aged mice show an accumulation of senescent p16*^Ink4a^*-positive microglia and oligodendrocyte progenitor cells (OPCs) (Ogrodnik et al., 2021). Single-cell analysis of the senescent microglial population demonstrates increased expression of IGF-1, MIF, IL-1β, and TIMP-2 (Ogrodnik et al., 2021). Notably, the dentate gyrus of female mice shows a stronger presence of senescent microglia and macrophages compared to male. The presence of these senescent myeloid cells in the dentate gyrus coincides with the downregulation of mediators of synaptic transmission and infiltration of T-cells and B-cells (Zhang X. et al., 2022). Whole-body clearance of SCs either genetically (AP20187 in *INK-ATTAC* mice) or pharmacologically (dasatinib + quercetin) significantly alleviated age-associated cognitive function in naturally aged 25–29-month-old mice (Ogrodnik et al., 2021). Furthermore, SC depletion with these strategies significantly reduced microglial activation and decreased peripheral T-cell presence into the aged hippocampus (Ogrodnik et al., 2021; Zhang X. et al., 2022). Hence, clearing SCs in the aged brain may rejuvenate the immune landscape of the hippocampus and ameliorate age-related cognitive decline.

### Impaired neurogenesis

Neurogenesis is the process of generating new neurons in the brain and is a relatively recent discovery in humans (Eriksson et al., 1998). The dentate gyrus of the hippocampus and lateral subventricular zone (SVZ) act as the neurogenic niches in the adult animal brain. Neurogenesis in the SVZ is linked to olfaction, while neurogenesis in the dentate gyrus contributes to learning and memory (Kumar et al., 2019).

With aging, lamin-B1 is downregulated in the outer granule cell layer of the dentate gyrus, which is mainly composed of neurons involved in the consolidation of memory (Matias et al., 2022). Furthermore, senescence in neuroblasts of the dentate gyrus in aged humans and mice results in the elaboration of a pro-inflammatory SASP that recruits and activates natural killer (NK) cells, which, in turn, eliminate neuroblasts (Jin et al., 2021). Attenuation of NK cell accumulation preserves neurogenesis and improved cognitive function in aged mice (Jin et al., 2021). Apart from neuronal senescence, Miranda et al. showed that inoculating neuronal stem cells (NSCs) with conditioned media of aged astrocytes (isolated from 13-month-old mice) reduces their proliferation compared to conditioned media of young astrocytes isolated from 3-month-old mice (Miranda et al., 2012).

### Synaptic dysfunction

Synaptic dysfunction is fundamental to brain aging with or without neurodegeneration. Peri-synaptic processes of astrocytes enwrap synaptic structures to regulate many aspects of inter-neuronal communication, including synaptogenesis, synapse maintenance, and synapse elimination (Sofroniew and Vinters, 2010). These peri-synaptic processes of astrocytes particularly cover excitatory synapses, where they take up glutamate in the synaptic cleft via transporters like GLT-1 and GLAST to prevent excitotoxicity. Glutamate is then converted to glutamine and supplied to neurons (Tani et al., 2014).

Astrocytes are reported to undergo senescence prematurely in response to ionizing radiation and hydrogen peroxide (H_2_O_2_)—termed stress-induced premature senescence (SIPS) (Cohen and Torres, 2019). SIPS astrocytes downregulate glutamate transporters and promote excitotoxicity (Limbad et al., 2020). It is important to state that the inducing stimulus has a profound impact on the specific phenotype of SCs. Hence, this section discusses the impact of aged/senescent astrocytes on synaptic function and not the consequences of SIPS astrocytes (for more detail on SIPS astrocytes see Cohen and Torres, 2019; Matias et al., 2022).

Astrocytes cultured *in vitro* to replicative senescence or isolated from senescence-accelerated mouse models demonstrate a decrease in their synaptogenic and neuroprotective capacity (Pertusa et al., 2007; García-Matas et al., 2008; Clarke et al., 2018; Matias et al., 2022). Indeed, co-culturing neurons with aged astrocytes that upregulate senescence markers is associated with increased neuron death (Pertusa et al., 2007; García-Matas et al., 2008). Furthermore, inoculating hippocampal neurons with replicative senescent astrocyte attenuates the release of the neurotransmitter glutamate from their presynaptic terminals, reflecting a decreased pool of synaptic vesicles (Kawano et al., 2012). However, how age-related astrocyte senescence impacts excitatory neurotransmission is conflicting. Studies have demonstrated both an increase and decrease in glutamate uptake in cortical and hippocampal senescent astrocytes of aged mice (García-Matas et al., 2008; Cao et al., 2019; Roalf et al., 2020; Matias et al., 2023). Similarly, expression levels of glutamate transporters in senescent astrocytes have been shown to increase and decrease (Lalo et al., 2011; Matias et al., 2023).

These diverging phenotypes may be determined by the senescence-inducing stimulus. Indeed, SIPS astrocytes downregulate glutamate transporters and promote excitotoxicity (Bitto et al., 2010; Limbad et al., 2020), whereas senescent astrocytes in the physiologically aged mouse hippocampus increase glutamate uptake possibly as a protective mechanism against excitotoxicity (Matias et al., 2023). Studying the age-related changes in astrocyte morphology and physiology without the pretext of cellular senescence, an elegant study compared the morphology and physiology of astrocytes derived from the CA1 region of the hippocampus of young (3–4-month-old), adult (9–12-month-old), and old (20–24-month-old) mice (Popov et al., 2021). As astrocytes age, their fine processes which enwrap synaptic structures become atrophic. This is associated with a reduction in the uptake of extracellular potassium and glutamate with the latter consequently binding extra-synaptic NMDA receptors, thereby impairing long-term potentiation (LTP) in the hippocampus—a process underlying synaptic plasticity responsible for learning and memory (Popov et al., 2021).

It is important to identify the specific triggers and mechanisms that drive aged astrocytes to seemingly divergent phenotypes regarding glutamate homeostasis, and whether these trajectories can be therapeutically manipulated to ameliorate age-related cognitive impairment. Whether cellular senescence figures in this process is also important to determine. Currently, despite cellular senescence being well-characterized in the context of SIPS astrocytes, its relevance in the aging process in the context of synaptic dysfunction is unclear.

### Glial heterogeneity and senescence

Cellular heterogeneity at the metabolomic, epigenomic, transcriptomic, and proteomic levels is being increasingly appreciated in astrocytes and microglia (Shafqat et al., 2023). Current consensus from the scientific community advocates moving away from the binary classification of astrocytes and microglia into pro-inflammatory (A1 or M1) and anti-inflammatory (A2 or M2) in favor of a spectrum of glial cell reactivity that ranges between the extremes of pro-inflammatory/neurotoxic and anti-inflammatory/neuroprotective phenotypes (Escartin et al., 2021; Paolicelli et al., 2022). For instance, the functions of the so-called A1 and A2 genes in astrocytes are mostly unknown, most astrocytes with aging and disease exhibit a mixed A1/A2 phenotype (Clarke et al., 2018; Grubman et al., 2019; Al-Dalahmah et al., 2020), and both pathological and protective clusters of astrocytes can co-exist in CNS diseases like multiple sclerosis and AD (Wheeler and Quintana, 2019; Jiwaji et al., 2022). Where astrocytes exist on this reactivity spectrum in different CNS disease states was recently shown to be determined by a core set of 61 transcriptional regulators (Burda et al., 2022), so it would be worth exploring how age-related astrocyte senescence alters these transcription factors to modulate astrocyte reactivity.

The binary classification of microglia into M1 and M2 is an *in vitro* classification based on the observation that microglia adopt diverging phenotypes when stimulated by lipopolysaccharide/interferon-γ and IL-4, respectively. However, this is not reflective of the *in vivo* reality in the healthy and diseased CNS, which contain a mixture of M1- and M2-promoting factors that are spatiotemporally dynamic (Ransohoff, 2016). Accordingly, microglia exhibit tremendous transcriptional heterogeneity, which is contingent upon their location in the brain and local environmental signals such as molecules released by astrocytes or BBB disruption in the context of aging (Zheng et al., 2021; Masuda et al., 2022). In healthy and disease CNS states, microglia exhibit a mixed M1/M2 gene signature (Morganti et al., 2016; Masuda et al., 2019), likely determined with their micro-environment that creates a spectrum of microglial activation (Xue et al., 2014). With aging, transcriptional microglial diversity decreases, but region-specific signatures are retained (Masuda et al., 2019). Gender also plays a key role in determining microglial state (Guneykaya et al., 2018; Lynch, 2022). With data showing that senescence in the aging brain is region-and gender-specific (Zhang X. et al., 2022), whether baseline microglial heterogeneity contributes to differential senescence susceptibility and/or senescence phenotypes based on gender or location in the brain is an outstanding question. Distinct microglial states appear with aging like lipid droplet-accumulating microglia (LDAMs) (Marschallinger et al., 2020) and white matter-associated microglia (WAMs) (Safaiyan et al., 2021), but whether age-associated senescent microglia constitute a distinct subset entirely or are a part of one of these other subsets remains to be determined. Furthermore, when considering senescence in myeloid cells, it is also not known whether senescence affects the CNS-associated macrophages (CAMs) [also called border-associated macrophages (BAMs)] that inhabit the perivasculature, choroid plexus, and meninges.

## Genetics of brain aging and cellular senescence

Brain aging is incredibly heterogenous and its consequences with respect to cognitive decline and neurodegeneration are not experienced uniformly. From a biological viewpoint, this may indicate that the susceptibility of the brain to cellular senescence may differ between individuals.

Between-individual differences in susceptibility to aging and neurodegenerative disease may be underpinned by the presence or absence of certain genetic variants. For example, the International Genomics of Alzheimer’s Disease Project conducted a meta-analysis of genome-wide association studies to extend the list of genetic variants associated with AD to more than 25 loci (Kunkle et al., 2019; Andrews et al., 2020). Genome-wide association studies have also identified several genetic variants associated with brain aging, highlighting that brain aging is influenced by the interaction of multiple genes with varying functionalities (Hammond et al., 2019b; McQuade and Blurton-Jones, 2019; Kim et al., 2023).

### TREM2

Many genetic variants associated with cognitive decline and AD are expressed in microglia, such as triggering receptor expressed on myeloid cells-2 (TREM2) (Jonsson et al., 2013). TREM2 appears to facilitate the pro-inflammatory disease-associated microglia (DAM) phenotype that appears in various CNS disease states (Keren-Shaul et al., 2017). It was recently shown that TREM2-expressing senescent microglia accumulate in aged and AD mouse brains, which share significant transcriptomic overlap with ‘highly activated microglia’, a subset that uniquely appears in the brains of aged mice and may trigger neuroinflammatory responses (Rachmian et al., 2023). Depleting TREM2-positive senescent microglia in 5xFAD mice (which are models of accelerated Aβ accumulation and AD) significantly improved cognitive status and decreased levels of inflammatory cytokines in the brain (Rachmian et al., 2023).

### Apolipoprotein-E4

The apolipoprotein-E4 (ApoE4) allele is the most well-known genetic variant associated with AD risk (Safieh et al., 2019). ApoE4 is associated with neuro-inflammaging, a decrease in proteins involved in synaptic plasticity and function, and BBB disruption particularly in the hippocampus (Halliday et al., 2016; Dai et al., 2018; Montagne et al., 2020). Broadly speaking, these changes are the same as those seen in the aging process. The well-established link between ApoE4 and neuro-inflammaging is reviewed elsewhere (Kloske and Wilcock, 2020). In terms of biological aging, ApoE4 expression is both a cause and consequence of mitochondrial dysfunction in the brain (Area-Gomez et al., 2020; Junxiang et al., 2020; Schmukler et al., 2020; Wynne et al., 2023), which is implicated in AD pathogenesis and in cellular senescence (Misrani et al., 2021; Miwa et al., 2022).

At the cellular level, ApoE4 exaggerates microglial pro-inflammatory responses to Aβ in AD mouse models (Kloske et al., 2021; Serrano-Pozo et al., 2021). ApoE4 leads to an enrichment of a specific microglial subset in normally aged mouse brains without AD/neurodegeneration (Lee et al., 2023). These ‘cluster 6 microglia (Mi_6)’ display a striking transcriptomic similarity to pro-inflammatory DAMs that appear in neurodegenerative disease (Keren-Shaul et al., 2017; Victor et al., 2022; Lee et al., 2023). ApoE, irrespective of variant, is believed to play a role in microglial ‘priming’ in early-stage AD by directing their activation towards a DAM phenotype that phagocytose tau-expressing neurons, which, in turn, induces senescence and type I IFN production that is toxic to synapses (Lau et al., 2023). Hence, ApoE4 may indirectly accelerate microglial senescence.

Separate from microglia, ApoE4 was recently found to decrease acetyl-CoA levels in hippocampal neurons of elderly ApoE4 mice, resulting in cellular senescence (Lv et al., 2023). Supplying glycerol triacetate (GTA) to increase acetyl-CoA levels to ApoE4 elderly mice decreased cellular senescence in neurons and increased expression of proteins related to synaptic plasticity.

### Repressor element-1-silencing transcription factor

Repressor element-1-silencing transcription factor (REST) expression in the brain is associated cognitively healthy brain aging but is lost in mild cognitive impairment and neurodegeneration (Lu et al., 2014; Zullo et al., 2019). Functionally, REST appears to protect neurons from oxidative stress and Aβ-related neurotoxicity and repress genes associated with AD (Lu et al., 2014). In animals and humans with extended longevity, REST is upregulated and downregulates neuronal excitability in the cerebral cortex (Zullo et al., 2019).

Recently, a GWAS implicated REST and its extended gene network with cognitive decline (Wang et al., 2023), indicating that perhaps genetic variations of REST could account for the between-individual variability in the brain aging phenotype. An obvious area of mechanistic exploration would be how REST influences biological aging hallmarks such as cellular senescence. Neurons isolated from REST-*knockout* mice demonstrate impaired autophagy, loss of proteostasis, higher oxidative stress, mitochondrial dysfunction and cellular senescence (Rocchi et al., 2021). Notably, restoring autophagy by treating neurons from REST-depleted mice with rapamycin reverses the senescence induction as well as its cellular hallmarks (Rocchi et al., 2021). These findings suggest that REST protects against neuronal senescence by maintaining autophagic flux in neurons.

## Epigenetics of brain aging and cellular senescence

Epigenetic modifications like DNA methylation, histone tail modifications, and microRNAs regulate gene expression without changing the genome sequence (Bonasio et al., 2010). The epigenome of humans is incredibly dynamic and undergoes consistent changes in response to environmental exposures as well as the aging process. In the brain, age-related epigenetic changes regulate the expression of genes involved in synaptic plasticity, learning and memory, and neurogenesis (Miller et al., 2010; Barter and Foster, 2018). This topic has been reviewed in detail elsewhere (Barter and Foster, 2018; Harman and Martín, 2020; Bacon and Brinton, 2021).

The consistent nature of DNA methylation alterations in whole blood and tissues has enabled the development of epigenetic clocks that leverage these patterns to precisely calculate an individual’s chronological and/or biological age (Horvath and Raj, 2018). The Horvath and Hannum clocks predict chronological age (Hannum et al., 2013; Horvath, 2013), the PhenoAge clock predicts phenotypic age and GrimAge is predictive of lifespan and mortality risk (Levine et al., 2018; Lu et al., 2019). The observation that tissues exhibit differential susceptibility to epigenetic aging has led studies to create clocks trained on methylation signatures of a specific tissue. For instance, a cortical clock trained on DNA methylation data from the human cortex spanning ages 1–108 years accurately predicted cortical age in a validation dataset (Shireby et al., 2020). A higher biological compared to chronological age as measured by epigenetic clocks has been termed epigenetic age acceleration (EAA) (Horvath and Raj, 2018). Epigenetic age and EAA are significantly more accurate than chronological age at predicting age-related atrophic changes of several regions of the cerebral cortex (Proskovec et al., 2020; Cheong et al., 2022; Hoare et al., 2022), rationalizing the use of epigenetic clocks as potential biomarkers of accelerated cortical aging and cognitive decline risk.

The observation that cellular senescence is associated with drastic changes in chromatin organization and transcriptomic and proteomic identity of a cell has led studies to characterize the epigenome of SCs. A detailed discussion on the broad epigenetic modifications displayed by SCs is beyond the scope of this review and have been detailed elsewhere (Zhu et al., 2021; Crouch et al., 2022). Suffice it to say that the specific epigenetic alterations seen in senescent cells are context-dependent, i.e., based on the type of senescent-inducing stimulus (e.g., replicative senescence vs. SIPS) (Shock et al., 2011; Chandra et al., 2012; Sakaki et al., 2017). Accordingly, not all types of cellular senescence are associated with epigenetic aging as measured by the aforementioned clocks; while replicative and oncogene-induced SCs are accompanied by epigenetic aging as measured by the Horvath clock, radiation-induced SIPS is not (Lowe et al., 2016).

Epigenetic alterations in SCs are particularly important for the expression of the SASP. Expression of high-mobility group box-2 (HMGB2) in SCs prevents the association of SASP genes into heterochromatin (Aird et al., 2016). Activating histone tail modification marks like acetylation and methylation are increased at key SASP genes in SCs (Capell et al., 2016; Tasdemir et al., 2016). At the same time, the activity of NAD-dependent histone deacetylase (HDAC) sirtuin-1, which can deacetylate and thereby decrease the expression of SASP genes, is decreased in SCs (Hayakawa et al., 2015; Wiley et al., 2016). KDM4, a histone deacetylase is activated in SCs secondary to genotoxic stressors like cancer therapy, leading to significant chromatin reorganization with increased expression of SASP proteins (Zhang et al., 2021). Targeting KDM4 in SCs attenuates the SASP without affecting cell-autonomous senescence, improving response to chemotherapy, and increasing survival in animals (Zhang et al., 2021). Given the essential nature of cellular senescence in wound healing and repair, maintaining cell cycle arrest in tumor cells, and embryogenesis, it may be more favorable to target the SASP rather than cell-autonomous senescent phenotype itself. However, epigenome alterations in the context of senescence in the aging brain are largely unexplored.

Nativio et al. (2018) showed that cognitively healthy elderly individuals age differently at the epigenetic level than patients suffering from neurodegenerative diseases like AD. The histone acetylation mark H4K16ac shows a genome-wide increase in the brain of elderly individuals who are cognitive normal, whereas it decreases in those with AD. The specific regions of DNA that show H4K16ac alterations in AD patients overlap significantly with known genetic variants that confer an increased risk of AD on GWAS (Nativio et al., 2018). Other studies have shown that an increase in H3K16ac marks is related to senescence induction and increased expression of proteins involved in the senescent phenotype (Dang et al., 2009; Rai et al., 2014). By this way, normal brain aging and cellular senescence seem to overlap in their epigenetic changes. Indeed, clustering specific genes showing H4K16ac changes during cognitively normal aging are also shown to be altered in SCs (Nativio et al., 2018). At the same time, AD appears to not be a consequence of normal aging and age-related senescence but rather a feature of dysregulated molecular aging. These findings also indicate that cellular senescence in the brain is not a ubiquitously pathological process that leads to neurodegenerative disease but rather a process of normal brain aging.

An example of how histone PTMs in the brain are potentially modifiable by environmental exposure and may be related to senescence is the neurotoxin paraquat, a risk factor for Parkinson’s disease (PD). In terms of its mechanism of action, paraquat increases histone acetylation and decreases activity of HDAC4 (Song et al., 2011). Exposing astrocytes to paraquat *in vitro* leads to HDAC4 inhibition-dependent senescence (Chinta et al., 2018). Furthermore, the burden of senescent astrocytes is increased in post-mortem brain tissue of PD patients, suggesting that epigenetic modifications in the brain are modifiable by environmental factors, which may then exert their downstream effects through cellular senescence.

## Cellular senescence in early brain aging

Accelerated aging-like states are a well-established phenomenon in several patient populations. These individuals prematurely develop age-related diseases including cognitive impairment, hinting toward the acceleration of biological aging processes such as cellular senescence in the brains of these patients. Evidence for accelerated brain aging in these patients is presented in Table 1, while Table 2 links the premature brain aging phenotype in these patients to cellular senescence. The direct role of senescence in CKD-and OSA-associated accelerated brain aging has not been demonstrated, hence we have restricted Table 2 to important studies on TBI, cancer therapy, and obesity.

**Table 1:** Evidence on early brain aging in certain patient populations.

**Patient population**	**Evidence about premature brain aging**
**Cancer survivors**	Cancer survivors overall and survivors of cancer for ≥5 years before cognitive testing, respectively, were significantly more likely than their co-twin to develop cognitive dysfunction (OR = 2.10, 95% CI = 1.36 to 3.24; *p* < 0.001) and (OR = 2.71, 95% CI = 1.47 to 5.01; *p* < 0.001) (Heflin et al., 2005).
	At a mean of 12 years after treatment for low-grade gliomas, patients who received radiotherapy had significantly worse attention deficits—even supposed safe fraction doses (≤2 Gy)—than those who did not receive radiotherapy (Douw et al., 2009).
**Traumatic brain injury**	TBI is associated with increased dementia risk (hazard ratio (HR) = 1.46; 95% CI = 1.41–1.52). Moderate/severe TBI increased risk of dementia across all ages (age 55–64: HR = 1.72; 95% CI 1.40–2.10 vs. age 65–74: HR = 1.46; 95% CI 1.30–1.64), whereas mild TBI may be more detrimental with increasing age (age 55–64 HR = 1.11; 95% CI 0.80–1.53 vs. age 65–74 HR = 1.25; 95% CI 1.04–1.51) (Gardner et al., 2014).
	A recent study examined the impact of mild TBIs on brain aging in 133 participants aged 20–83 years using T1-weighted magnetic resonance (MRI) imaging findings taken within 7 days and 6 months after TBI. This study showed that mild TBI ages the brain of older adults (>60 years old) by approximately 10 years. Biologically, hallmarks of CNS aging such as neuroinflammation, glial cell reactivity, excitotoxicity, and abnormal protein accumulation—tau and amyloid-beta (Aβ)—have been observed in TBI brains (Halstrom et al., 2017).
**Obesity**	Obesity increases the risk of mild cognitive impairment, independent of age and cardiovascular risk factors. Obesity in middle-aged adults increases risk of dementia by 1.5–2 times up to the age of 65 (Dahl et al., 2013; Hassing et al., 2010; Whitmer et al., 2008).
	Obesity has been shown to double the risk of Alzheimer’s disease (Anstey et al., 2011; Pedditzi et al., 2016), with postmortem studies of obese elderly individuals showing increased concentrations of amyloid-beta and hyperphosphorylated tau, which were associated with hippocampal atrophy (Mrak, 2009).
**Obstructive sleep apnea**	Compared to controls, patients with moderate–severe OSA performed worse on the Rey Auditory-Verbal Learning test (immediate and delayed recall), Stroop test, and Digit span backward scores. Patients with moderate–severe OSA had a lower volume of cortical gray matter (GM), right hippocampus, and the right and left caudate (Torelli et al., 2011).
	Greater OSA severity, as indicated by a higher AHI score and lower oxygen saturation during sleep, is directly correlated with the promotion of several biological aging hallmarks in patients younger than 50 years old, including altered cellular communication, dysregulated nutrient sensing, mitochondrial dysfunction, and genomic instability (Pinilla et al., 2021).
**Chronic kidney disease**	A meta-analysis of cross-sectional and longitudinal studies, respectively, of 54, 779 CKD patients demonstrated that patients with CKD were significantly more likely to suffer cognitive decline than patients without CKD (OR 1.65, 95% CI 1.32–2.05; *p* < 0.001 and OR 1.39, 95% CI 1.15–1.68; *p* < 0.001, respectively) (Etgen et al., 2012).

**Table 2:** Evidence of cellular senescence in early brain aging.

**Patient population**	**Evidence of cellular senescence in early brain aging**
**Traumatic brain injury**	Subjecting young (12-week-old) and aged (18-month-old) male C57BL/6 mice to controlled cortical impact (CCI) results in more severe deficits in forelimb grip strength, balance, motor coordination, spontaneous locomotor activity, and anxiety-like behavior in the aged group. Aged mice showed higher number of peripheral leukocyte infiltration post-TBI. Microglia of aged mice showed significantly higher senescent markers (BCL-2, p16^*Ink4a*^, p21^^Cip1/Waf1^^, and H2AX) at baseline and after CCI, associated with impairment in phagocytosis and higher IL-1β production (Ritzel et al., 2019).
	Mild-repeated TBI (mrTBI) in C57BL/6 mice results in behavioral disinhibition and cognitive impairment. Examination of the brain 1-week post-TBI reveals upregulation of DNA damage, the cytosolic DNA sensor cGAS/STING, and cellular senescence. ScRNA-seq revealed distinct neuronal clusters, astrocytes, OPCs, immune cells, and vascular cells showing features of cell cycle arrest, SCAP upregulation, and SASP signaling. Treating mrTBI mice with navitoclax decreased senescence markers in male mice but not female mice. Navitoclax treatment improved cognitive performance on the Morris water maze test in male mice (Schwab et al., 2021, 2022).
**Cancer survivors**	Brain tissues of irradiated cancer patients show an upregulation of p16^*Ink4a*^-positive astrocytes compared to age-matched controls and untreated cancer patients. Irradiated astrocytes *in vitro* downregulate glutamate transporters and produce IL-6. In human astrocytes, Δ133p53 prevents radiation-induced senescence and ameliorates astrocyte-mediated neuroinflammation and neurotoxicity (Turnquist et al., 2019; Limbad et al., 2020).
	Paclitaxel (PTX) induces senescence (SA β-gal) in cebromicrovascular capillary endothelial cells of transgenic p16-3MR mice. PTX treatment-related senescence is associated with reduced indices of cerebrovascular density, deficits in neurovascular coupling responses, greater transcellular permeability between cerebromicrovascular endothelial cells, and significant impairments in cognitive function and spatial memory. Ganciclovir-or ABT263-mediated clearance of SCs in PTX-treated pmice significantly improved cognitive function, neurovascular coupling, and BBB integrity (Ahire et al., 2023).
**Obesity**	HFD in C57BL/6 mice leads to anxiety-like behavior. Genetic clearance of SCs by AP20187 in INK-ATTAC or dasatinib + quercetin in leptin receptor-deficient obese mice both reduced anxiety-like behavior. SCs accumulated in the amygdala and hypothalamus (but not in the cortex or hippocampus), which was significantly decreased by AP20187. HFD increased accumulation of lipid droplets in senescent astrocytes and microglia in periventricular regions, skewing them toward inflammation with higher CXCL1 and IL-6 production. Obese mice displayed significant reductions in the numbers of NSCs, immature neurons, and CD133^+^ ependymal cells in the lateral SVZ, indicating impaired neurogenesis. AP20187 significantly increased stem cell and astrocyte numbers in the SVZ, indicating increased neuronal plasticity post-treatment (Ogrodnik et al., 2019).

### Traumatic brain injury

Mild TBIs, including concussions or other minor sub-concussive head trauma, are common in the general population. All forms of TBI regardless of severity confer a higher long-term risk of dementia (Gardner et al., 2014). TBI is also associated with an increased long-term risk of developing early-onset AD, PD, and chronic traumatic encephalopathy (Nambiar et al., 2023).

Senescence markers like SA-β-gal, reduced lamin-B1, γ-H2AX, and SASP components IL-1β, IL-6, CXCL1, and CCL8 are elevated in the brains of repeated TBI mouse models displaying cognitive deficits and in post-mortem brain samples of individuals with a history of repeated concussions (Tominaga et al., 2019; Schwab et al., 2019a,b; Mester et al., 2021). TBI acutely induces oxidative stress and DDRs in the brain (Singh et al., 2006; Czarny et al., 2015; Halstrom et al., 2017). Indeed, markers of DDR are significantly elevated 24 h after injury, while gene expression profiles at 7 and 14 days post-injury are consistent with cellular senescence and the production of a pro-inflammatory SASP (Schwab et al., 2021). Aged mice display worse motor and cognitive outcomes post-TBI than younger mice, associated with a higher senescence burden in the cortex and in microglia at baseline that is significantly exacerbated by the traumatic insult (Ritzel et al., 2019). This microglial phenotype of aged mice exhibits diminished homeostatic functions and skewing towards inflammation, which may explain why aged mice develop more pronounced neuroinflammation post-TBI (Ritzel et al., 2019). These findings suggest that a higher burden of SCs may explain why older age is a poor prognostic factor for TBI.

Abnormal protein accumulation is a pathologic hallmark of TBI. Like AD, TBI features Aβ accumulation, which has been found to trigger senescence in astrocytes (Bhat et al., 2012; Shang et al., 2020), microglia (Flanary et al., 2007), and NSCs (Wei et al., 2016; Scopa et al., 2020; Ohline et al., 2022). Depleting senescent OPCs with the senolytic cocktail of dasatinib and quercetin reduces Aβ load, alleviates neuroinflammation, and improves cognitive function (Zhang et al., 2019). TBI also promotes the intracellular accumulation of hyperphosphorylated tau in neurofibrillary tangles (NFTs) in neurons (Katsumoto et al., 2019; Edwards et al., 2020). In AD, NFT-containing neurons upregulate p16*^Ink4a^* and pro-inflammatory SASP, associated with cerebral atrophy and cognitive decline (Musi et al., 2018). The senolytic cocktail of dasatinib and quercetin significantly reduces the burden NFT-containing cortical neurons, decreasing cerebral atrophy and suppressing pro-inflammatory SASP expression (Musi et al., 2018). Senescent astrocytes and microglia also accumulate in mouse models of tauopathy, promoting cortical degeneration and cognitive dysfunction (Bussian et al., 2018). Tau-induced senescent astrocytes contribute to cognitive decline via HMGB1 and NLRP3 inflammasome activation (Gaikwad et al., 2021). Admittedly however, prominent distinctions between the pathophysiology of AD and TBI are evident (Katsumoto et al., 2019). For instance, cellular senescence appears to be an acute consequence of TBI which precedes pathologic protein, but is a consequence of Aβ and hyperphosphorylated tau in AD (Schwab et al., 2021).

Repeated mild TBI is also associated with the emergence of senescent astrocytes and neurons in the cortex and hippocampus (Schwab et al., 2022). Single-cell RNA-sequencing of neurons revealed several transcriptionally distinct neuronal clusters all of which upregulate core features of cellular senescence including p16*^Ink4a^*/p21*^Cip1/Waf1^*, SCAPs, and a pro-inflammatory SASP. The transcriptome of astrocytes indicated the production of a pro-inflammatory SASP and dysregulated glutamate neurotransmission with downstream excitotoxicity, indicating the TBI serves as a type of SIPS stimulus in astrocytes with downregulation of glutamate transporters (Schwab et al., 2022). Administration of the senolytic ABT263 significantly decreases p21*^Cip1/Waf1^* expression and improves memory and executive function in male mouse models of repeated mild-TBI (Schwab et al., 2022).

### Cancer survivors and therapy-induced senescence

Adult and childhood cancer survivors experience accelerated aging manifested in the premature onset of frailty, sarcopenia, osteoporosis, osteoarthritis, second cancers, endocrinopathies, and other chronic diseases earlier than age-matched healthy controls (Ness et al., 2015; Cupit-Link et al., 2017; Shafqat et al., 2022).

Cancer treatment-related cognitive impairment is well-documented and affects patients regardless of cancer type and location (Lange et al., 2019; Országhová et al., 2021; Table 1). Cancer treatments such as chemotherapy, radiotherapy, hormonal therapy, and immunotherapy induce senescence in tumor cells but also in normal cells, known as therapy-induced senescence (TIS) (Wang et al., 2020b). Radiotherapy exposure triggers a DDR that promotes apoptosis or senescence, both of which would arrest tumor growth. However, this phenomenon occurring in non-tumor cells leads to systemic SC accumulation and subsequent tissue aging (Wang et al., 2020b).

Radiation-induced brain injury is a major risk factor for long-term neurocognitive dysfunction in cancer survivors (Greene-Schloesser et al., 2012). One-year-old mice exposed to full-body radiation exhibit a significantly higher burden of senescent neurons in the hippocampus comparable to levels in two-year-old mice, which is associated with cognitive decline (Fielder et al., 2022). Similarly, the irradiated brain tissue of cancer patients exhibits a significantly higher burden of p16*^Ink4a^*-positive senescent astrocytes than age-matched controls and patients with the same cancer type who did not receive radiation (Greene-Schloesser et al., 2012; Turnquist et al., 2019), although whether the irradiated cancer group demonstrated a greater burden of neurocognitive deficits was not determined. Irradiated human senescent astrocytes downregulate glutamate transporters that lead to excitotoxicity (Limbad et al., 2020). Furthermore, co-culturing NSCs with irradiated human senescent astrocytes decreases NSC viability and increases their apoptosis, mediated by astrocyte-derived IL-6 (Turnquist et al., 2019). Reversing astrocyte senescence attenuates astrocyte-mediated neuroinflammation, indicated by reduced IL-6 production, and increases mRNA expression of the neurotrophic factor IGF-1 (Turnquist et al., 2019). Administering senolytic drugs (navitoclax or dasatinib+quercetin) or senomorphic drugs (metformin) to sub-lethally irradiated mice effectively prevents frailty progression, improves muscle and liver function, and improves short-term memory, although the exact brain cell types targeted by these medications which resulted in improved memory were not determined (Fielder et al., 2022). Nevertheless, emerging evidence suggests that cellular senescence could be a novel therapeutic target for preventing radiotherapy-related cognitive deficits in cancer survivors.

Chemotherapy-induced cognitive impairment (CICI) is a well-known phenomenon that affects long-term outcomes of cancer survivors, encompassing deficits in memory, executive function, attention, processing speed, and psychomotor dysfunction (Ossorio-Salazar and D’Hooge, 2023). With respect to chemotherapy, Demaria et al. reported that doxorubicin-treated mice have systemic upregulation of senescence markers and SASP components like IL-1, IL-6, MMP-3/9, CXCL1, CXCL10, and CCL20 (Demaria et al., 2017). It is remarkable that depleting SCs almost entirely prevents doxorubicin-induced cardiomyopathy and significantly increases the nocturnal running time of mice (Demaria et al., 2017). Similarly, the targeted apoptosis of SCs mitigates doxorubicin-induced hepatotoxicity and improves fitness, fur density, and renal function of prematurely aged and normally aged mice (Baar et al., 2017).

Paclitaxel is a notorious chemotherapeutic agent known to cause CICI (Ahire et al., 2023). Treating transgenic p16-3MR mice with paclitaxel for 10 days resulted in cerebrovascular endothelial cell senescence (SA-β gal-positivity), which was associated with deficits in spatial memory cognitive performance, a decrease in microvascular density, and an increase in BBB permeability and neuroinflammation. Importantly, genetically (ganciclovir) or pharmacologically (senolytic anti-BCL2 agent ABT263) depleting senescent endothelial cells in p16-3MR mice restored BBB integrity, decreased neuroinflammation, improved microvascular density, and improved cognitive performance (Ahire et al., 2023). These results, for the first time, made a strong case that senescence of cerebrovascular endothelial cells is involved in the pathogenesis of CICI and that senolytic treatment may be a novel intervention for ameliorating CICI.

### Obesity

Obesity negatively impacts cognition independent of its cardiovascular comorbidities and increases the lifetime risk of Alzheimer’s disease and dementia (Beydoun et al., 2008). Neuroimaging studies in obese patients have shown reduced cortical volume, particularly in areas mediating cognition such as the hippocampus (Ward et al., 2005; Raji et al., 2010; Table 1).

Mechanistically, obesity accelerates numerous biological aging processes, including inflammaging, oxidative stress, telomere attrition, epigenetic alterations, mitochondrial dysfunction, and cellular senescence (Nunan et al., 2022). A higher SC burden has been observed in adipose tissue in obesity, affecting preadipocytes, adipocytes, endothelial cells, and adipose tissue-resident macrophages (Smith et al., 2021). Furthermore, SCs accumulate in the kidneys of HFD-obese mice, which is associated with renal dysfunction (Kim et al., 2019). The livers of obese mice also display a higher SC burden, linked to hepatic steatosis and non-alcoholic fatty liver disease (Aravinthan et al., 2013; Ogrodnik et al., 2017).

However, evidence directly implicating senescence as a mechanism of obesity-related brain aging is currently limited. Ogrodnik et al. (2019) documented senescence in glial cells in the amygdala and hypothalamus in obese INK-ATTAC transgenic mice and leptin receptor-deficient obese mice, linked to anxiety-like behavior. Depleting SCs in obese INK-ATTAC transgenic mice decreased SC burden in the amygdala and hypothalamus and relieved anxiety-related behaviors. Obesity also leads to a decrease in the population of NSCs in the SVZ, which could be partially recovered by AP20187 treatment in INK-ATTAC mice (Ogrodnik et al., 2019). These results indicate that obesity-related senescence preferentially affects areas of the brain responsible for anxiety and fear like the amygdala, but not the hippocampus, which is responsible for learning and memory. Modulation of senescence may therefore hold therapeutic value in treating neuropsychiatric disorders in obese individuals.

### Obstructive sleep apnea

OSA is an increasingly prevalent sleep breathing disorder that results in apnea or hypopnea due to episodic partial or complete airway obstruction during sleep. Common symptoms of OSA include loud snoring, gasping, or choking during sleep, nighttime awakenings, daytime sleepiness, and fatigue (Young et al., 2004; Madani and Madani, 2009; Rundo, 2019). OSA patients, particularly those with moderate-to-severe disease, face an increased risk of cognitive impairment compared to age-matched healthy controls (Torelli et al., 2011). OSA patients also tend to develop chronic diseases earlier than healthy controls, including cardiovascular disease, metabolic disorders, cancer, and neurodegeneration (Veasey and Rosen, 2019). Biologically, OSA is independently linked to higher burdens of oxidative stress and inflammaging (Lavie, 2015; Liberale and Camici, 2020), prompting consideration of OSA as an accelerated aging phenotype (Gaspar et al., 2017).

OSA leads to chronic intermittent hypoxia (CIH) and sleep fragmentation (Gaspar et al., 2017). Sleep disturbance in OSA has been shown to accelerate telomere attrition, leading to replicative senescence (Tempaku et al., 2015; Turkiewicz et al., 2021). Exosomes derived from the blood of OSA patients can induce endothelial cell senescence and vascular dysfunction, which is partially reversible with continuous positive airway pressure (CPAP) therapy (Khalyfa et al., 2020). CIH has recently been demonstrated to induce senescence in multiple tissues, including preadipocytes, kidneys, vasculature, and heart (Polonis et al., 2020; Badran et al., 2021; Wei et al., 2022). In the brain, CIH increases markers of oxidative stress, DNA damage, and inflammation in several regions associated with early-stage AD and PD (the entorhinal cortex and substantia nigra, respectively). While this study did not evaluate markers of senescence, such an environment is known to induce senescence (Martínez-Cué and Rueda, 2020). However, no study so far has directly investigated the role of senescence in OSA-related brain changes.

### Chronic kidney disease

CKD affects about 15% of US adults and incurs significant morbidity, mortality, and health expenditures (Hoerger et al., 2015; Kovesdy, 2022). Chiu et al. (2019) demonstrated that non-demented end-stage kidney disease patients receiving dialysis exhibit structural and cognitive changes associated with normal aging. CKD patients are also susceptible to systemic early aging-related conditions, including osteoporosis and pathologic fractures (Pimentel et al., 2017; Hsu et al., 2020), hypogonadism (Skiba et al., 2020), impaired wound healing (Maroz and Simman, 2013), insulin resistance (Spoto et al., 2016), cardiovascular disease (Jankowski et al., 2021), cerebrovascular disease (Vanent et al., 2022), cognitive impairment (Etgen et al., 2012), immunosenescence (Crépin et al., 2020), and sarcopenia (Stenvinkel and Larsson, 2013).

Mouse models of CKD exhibit increased microglial activation, which correlates with incidence of cerebral microhemorrhages (Fang et al., 2023). Uremic toxins that accumulate in CKD, such as indoxyl sulfate, p-cresyl sulfate, trimethylamine-*N*-oxide (TMAO), and urea, increase BBB permeability (Lau et al., 2020; Fang et al., 2023). These changes—BBB leakiness, microglial activation, and neuroinflammation—are also observed in the aged brain (Sierra et al., 2007; Koellhoffer et al., 2017; Marschallinger et al., 2020; Paul et al., 2021; Connolly et al., 2022; Iwao et al., 2023). Thus, CKD may precipitate a premature aging phenotype by accelerating fundamental aging processes (Huang et al., 2022; Arabi et al., 2023).

Cellular senescence in CKD involves multiple mechanisms. Hyperphosphatemia induces senescence in endothelial cells, myoblasts, and vascular smooth muscle cells, contributing to vascular aging (Troyano et al., 2015; Yamada et al., 2015; Sosa et al., 2018; Liu et al., 2021). Uremic toxins in the bloodstream of CKD patients can induce senescence by imposing oxidative stress and consequent DNA damage (Vaziri, 2004; Han et al., 2018; Lee et al., 2018). For example, indoxyl sulfate and TMAO promote ROS-dependent senescence in the aorta, associated with endothelial dysfunction, vascular calcification, and wall stiffening—all indicators of vascular aging (Ke et al., 2018; Brunt et al., 2020, 2021; Lau et al., 2020). Indoxyl sulfate also induces senescence in CD34+ hematopoietic stem cells and curbs their differentiation into mature erythrocytes, possibly contributing to the normocytic normochromic anemia observed in CKD patients (Duangchan et al., 2022). *P*-cresyl sulfate promotes senescence features—ROS production, DDR, and proinflammatory SASP—in mouse adipocytes, promoting adipose tissue inflammation and insulin resistance (Koppe et al., 2013; Tanaka et al., 2020).

In the brain, indoxyl sulfate activates the aryl hydrocarbon receptor (AhR) on astrocytes and microglia, increasing oxidative stress and neuroinflammation and accelerating cognitive impairment (Adesso et al., 2017, 2018; Bobot et al., 2020). TMAO-dependent astrocyte reactivity is implicated in aging-related cognitive decline (Clarke et al., 2018; Csipo et al., 2020; Brunt et al., 2021). TMAO has been shown to induce senescence in hippocampal neurons, decreasing their expression of synaptic plasticity-related proteins (Li et al., 2018). DMB, which is an enzyme that decreases TMAO levels, mitigates cognitive decline in mouse models of accelerated cellular senescence (Lanz et al., 2022), potentially reflecting a reduction in SC burden in the brain of treated mice.

## The future of senotherapies

The remarkable evolution of senolytics and senomorphics from bench to bedside has resulted in much hype surrounding these drugs as so-called anti-aging agents. However, several unanswered questions must first be addressed to effectively translate these agents into clinical practice (Shafqat et al., 2022). Firstly, senescence induction plays essential roles in wound healing, embryogenesis, and tumor suppression, underscoring the importance of delineating potential contraindications. Secondly, the long-term effects of removing or altering SCs are unknown. In the context of brain aging, it is crucial to consider the possible long-term consequences on CNS function of depleting senescent neurons incapable of renewal. There is also no agreed-upon senescence biomarker, a composite score of markers, or a way to gauge the effectiveness of senolytics or senomorphics in humans other than observable phenotypic/functional improvement. Lastly, as of 2023, most data on senolytics and senomorphics come from preclinical animal studies. Data from ongoing clinical trials studying these drugs in larger and longer cohorts will provide more information on the efficacy and, more importantly, the safety of these medications.

Other senescence-targeting strategies, such as immune-direct strategies and hyperbaric oxygen therapy (HBOT) are currently under study and may provide an alternative to the SCAP-targeting senolytics or SASP-targeting senomorphics. Gene therapy and epigenetic reprogramming may also be feasible approaches to combat cellular senescence in aging and have been recently reviewed (Zhang et al., 2020; Wang et al., 2022; Yu et al., 2023).

### Immune-mediated SC clearance

'Senescence surveillance’ was introduced by Kang and colleagues when they observed that senescent pre-malignant hepatocytes secreted a SASP that recruited CD4^+^ T-cells, which cleared these SCs (Kang et al., 2011). Mechanistically, macrophages can upregulate class I MHC and antigen-processing machinery in senescent tumor cells, facilitating their recognition by CD8^+^ T-cells (Reimann et al., 2021; Sturmlechner et al., 2021; Chen et al., 2023; Marin et al., 2023). SCs also transfer antigenic peptides to dendritic cells, which, in turn, activate CD4^+^ T-cells (Chandra et al., 2012; Prata et al., 2018). The SC peptidome is significantly different from their parental non-senescent cells and can act as a target for cell-mediated (T-cell) and humoral (B-cell) immune responses (Frescas et al., 2017; Suda et al., 2021). One such protein termed a ‘seno-antigen’, glycoprotein nonmetastatic melanoma protein B (GPNMB), was formulated into a senolytic vaccine and injected into progeroid mice, effectively reducing SC burden, alleviating pathological effects of obesity and atherosclerosis, and extending lifespan (Suda et al., 2021). Amor et al. developed chimeric antigen receptor (CAR) T cells targeting urokinase-type plasminogen activator receptor to deplete SCs in mouse models of lung cancer and hepatic fibrosis (Amor et al., 2020). Given this apparent immunogenicity of SCs, it is curious why SCs accumulate in aged tissues and are not cleared by the immune system. An aging functionally declining immune system may not be as effective in clearing SCs (Prata et al., 2018), or SCs may upregulate certain surface proteins like PD-L1, which allow them to evade immune surveillance (Onorati et al., 2022; Wang et al., 2022). How immune-directed senolytic strategies can be repurposed to target SCs in the brain is unchartered territory.

### Hyperbaric oxygen therapy

Hyperbaric oxygen therapy (HBOT) involves breathing pure oxygen at a heightened atmospheric pressure to increase partial pressure of oxygen in the bloodstream and improve oxygen delivery to tissues. Oxygen has a dual relationship with aging (Hopf et al., 2005; de Wolde et al., 2022; Fu et al., 2022). It is the source of ROS that induce lipid peroxidation, DNA damage, and protein dysfunction. However, repeated exposures to high oxygen pressures, such as in HBOT, can augment antioxidant responses and angiogenesis and reduce inflammation (Hopf et al., 2005; de Wolde et al., 2022; Fu et al., 2022). These effects of HBOT are evident in tissue rejuvenation strategies, such as ischemic wound healing and recovery after muscle injury, where it decreases inflammation and apoptosis to promote healing (Zhang and Gould, 2014; Oyaizu et al., 2018).

As a senescence-alleviating treatment modality, HBOT can reduce the number of SA-β gal-positive cardiomyocytes in aging pre-diabetic rats (Bo-Htay et al., 2021). A prospective clinical trial with a cohort of 70 healthy participants (mean age = 68.07 years) receiving HBOT for 3 months reported a significant increase in collagen density, elastic fiber length, and vascularity in serial skin biopsies in treated individuals (Hachmo et al., 2021). Tissue SCs were also significantly decreased in the treated group, indicated by reduced lipofuscin expression (Hachmo et al., 2021). Other studies have documented a decrease in the levels of cytokines, chemokines, and MMPs after HBOT in the context of aging, indicating that this therapeutic modality can attenuate SASP production (De Wolde et al., 2021; Fu et al., 2022). Telomere elongation appears to be one of the primary mechanisms by which HBOT attenuates cellular senescence (i.e., replicative senescence). A study on deep sea divers and a prospective clinical trial of 35 old adults (>65 years old) receiving HBOT both documented significantly increased telomere length in peripheral blood mononuclear cells after the treatment (Shlush et al., 2011; Hachmo et al., 2020).

HBOT has also been applied to the context of cognitive decline. Chen and colleagues intraperitoneally injected d-galactose into mice to mimic age-related cognitive impairment and simultaneously administered HBOT, which significantly reversed d-galactose-induced learning and memory impairment and decreased p16*^Ink4a^*, p21^*Cip1/Waf1*^, and p53 expression in the hippocampus (Chen et al., 2016). Similarly, HBOT restores cognitive function in d-galactose and obese aged rats, associated with a decrease in SA-β-gal positive cells in the hippocampus (Shwe et al., 2021). HBOT also improves the cognitive function of TBI survivors (Biggs et al., 2021; Chen et al., 2022; Harch, 2022), at least partly underpinned by reduced neuroinflammation and MMP production, which may reflect an attenuated SASP (Vlodavsky et al., 2006).

## Conclusion and outlook

Emerging research underscores the critical involvement of cellular senescence in the process of brain aging. There is also mounting evidence from animal models that cellular senescence plays a significant role in the pathogenesis of premature brain aging.

Cancer survivors who have received chemotherapy or radiotherapy—a population which is increasing annually as well as getting older—are at an ever-increasing risk of morbidity related to cognitive decline. Therefore, increasing their representation in senotherapeutic clinical trials will be crucial. However, despite encouraging safety and tolerability results in phase 1 clinical trials, their efficacy in a host of chronic diseases must be evaluated before testing these drugs in frail and vulnerable populations like cancer survivors.

From a mechanistic viewpoint, we are still confronted with several gaps in our understanding. The upstream biological cues that regulate senescence of the aging brain, for instance, require further study. Secondly, to address why individual brains age at different rates, studies must directly investigate genetic and epigenetic regulation of senescence in the brain and its relationship to aging and cognition. Thirdly, understanding the specific mechanisms through which senescent brain cells contribute to neuropathology is still in its initial stages. Current strategies for modulating SCs in mouse models are systemic and lack specificity; to illustrate this, using AP20187 in INK-ATTAC transgenic mice systemically depletes all p16*^Ink4a^*-expressing cells, but not all cells expressing p16*^Ink4a^* are senescent and not all p16*^Ink4a^* -positive SCs are present in the brain. The question of whether the observed improvements in cognitive function and neuroinflammation after genetic or senolytic-mediated SC elimination in mice can be attributed to a reduction in senescent brain cells as opposed to peripheral effects is a critical distinction. Ogrodnik et al. (2019) addressed this issue by transplanting SCs peripherally in mice and evaluating whether this recapitulated senescence-related anxiety-like behaviors in obese mice. They also evaluated whether inhibiting circulating SASP factors implicated in neuropathology and depleting SCs in the brain achieve similar therapeutic benefits with respect to neurobehavioral outcomes. Nevertheless, experiments utilizing transgenic mice that allow selective depletion of SCs in the brain would be required to causally link senescence in specific brain cells to aging-related brain changes and better comprehend the neuroinflammatory dynamics in the aging brain.

Future research focusing on this pivotal area has the potential to uncover transformative therapeutic targets and strategies for alleviating brain aging. In doing so, we could dramatically lessen the substantial impact on patients, healthcare systems, and society at large.

## Author contributions

AS: Conceptualization, Writing – original draft, Writing – review & editing. SK: Writing – original draft. MO: Writing – original draft, Writing – review & editing. MN: Writing – original draft. IA: Writing – original draft, Writing – review & editing. KA: Writing – review & editing. AY: Writing – review & editing. TT: Writing – review & editing, Supervision. JK: Writing – review & editing, Supervision. SH: Conceptualization, Writing – review & editing.
